# Card-not-present fraud: using crime scripts to inform crime prevention initiatives

**DOI:** 10.1057/s41284-022-00359-w

**Published:** 2022-11-14

**Authors:** Amanda Bodker, Phil Connolly, Oliver Sing, Benjamin Hutchins, Michael Townsley, Jacqueline Drew

**Affiliations:** School of Criminology and Criminal Justice, Mount Gravatt Campus, Griffith University, Brisbane, QLD 4111 Australia

**Keywords:** Fraud, Card-not-present fraud, Crime script analysis, Crime prevention, Online retail

## Abstract

Growth in the online retail sector and improvements in card-present authorisation measures have led to substantial increases in card-not-present (CNP) fraud, particularly in the online retail sector. This article uses crime script analysis to understand the commission process of CNP fraud in online retail settings. Drawing upon previous crime script articles and industry reports we outline the steps involved in the three stages of CNP fraud: preparation; doing it; and getting away. From this script, points of disruption are identified and we discuss prevention measures that stakeholders such as businesses and financial institutions could implement to reduce bad actors opportunities for CNP fraud.

## Introduction

Online retail has been an area of consistent growth for the last decade. However, the COVID-19 pandemic triggered a rapid acceleration in e-commerce trends. In the United States (US), from 2009 to 2019, e-commerce penetration steadily increased 1% annually. In the first quarter of 2020 alone, with the advent of the pandemic, e-commerce increased 10%, demonstrating 10 years of growth in 3 months (McKinsey and Company 2020). This rapid acceleration in online retail growth has increased online fraud opportunities (ACFE 2021), particularly card-not-present (CNP) fraud.

**Fig. 1:**
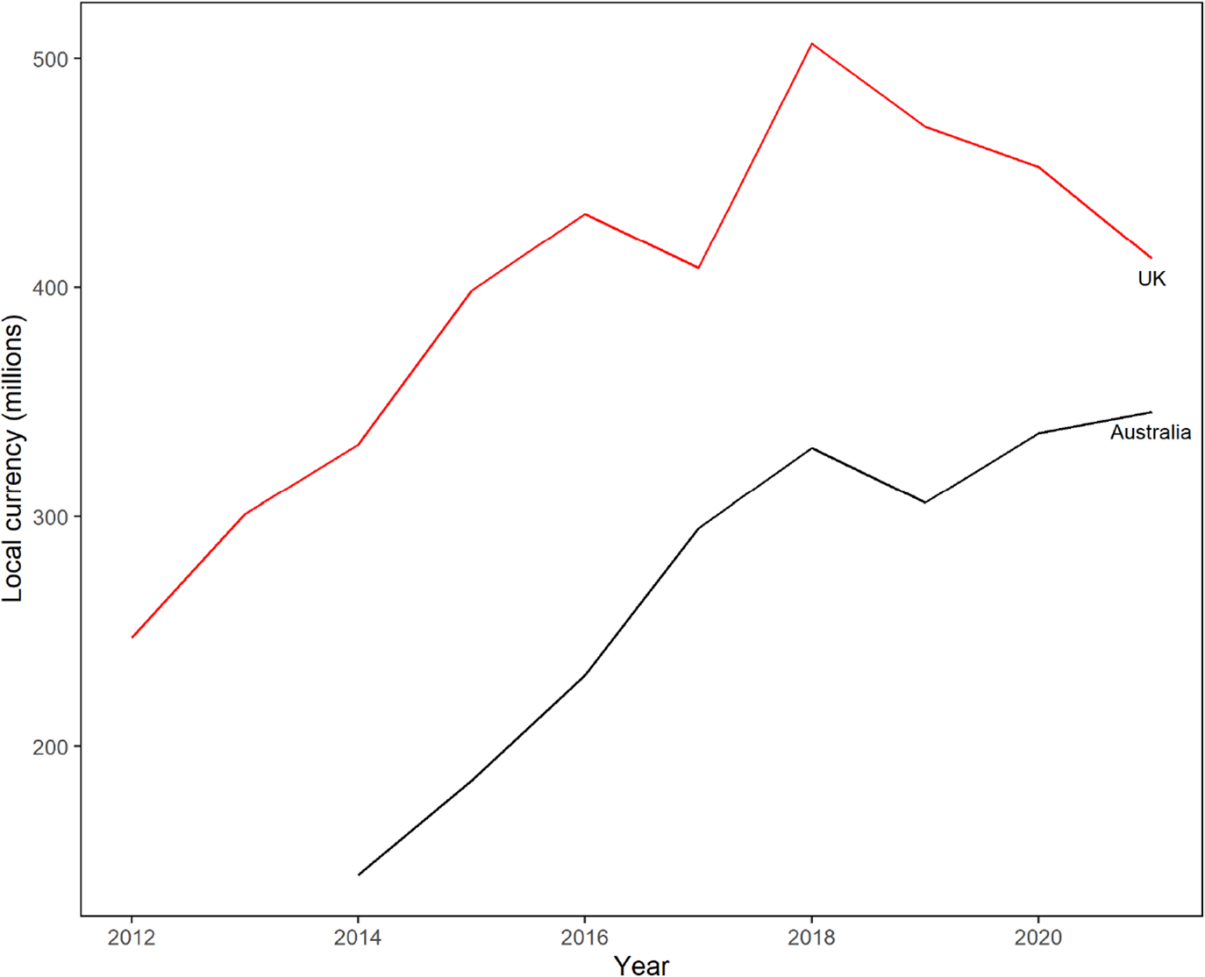
CNP fraud values (in GBP and AUD, respectively) between 2012 and 2021. *Sources* UK Finance (2022), Australian Payments Network (2020, 2022)

Card-not-present (CNP) transactions are those where the business does not witness the physical payment card, with online or telephone sales being the most prevalent examples (Australian Payments Network 2018). CNP fraud involves the illegal acquisition of the payment details of another individual and subsequent unauthorised use of this information for a CNP transaction. As payment card security measures have improved (smart cards, chip rather than magnetic stripe, etc.) some card-present fraud methods such as counterfeiting and mail interception have become more difficult (Webb 1996), with bad actors moving to other methods (Levi 2003). In the United States (USA), CNP fraud accounted for 61% of detected payment card frauds in 2016, costing $4.57B USD (Gerdes et al. 2018). In the United Kingdom (UK) CNP fraud has been the most common type of payment card fraud since 2014 (UK Finance 2020). CNP fraud has also consistently been the most prevalent form of payment card fraud in Australia since 2014 (Australian Payments Network 2020). In 2019, CNP fraud accounted for 85% of all detected payment fraud transactions that occurred in Australia, representing direct costs of $224 million AUD for Australian-issued cards and another $82 million on overseas-issued cards (Australian Payments Network 2020). This has been a persistent problem that has generally increased across the last decade. Figure 1 demonstrates this pattern for the UK and Australia, both of which have readily accessible and reliable information available.

This paper draws on academic articles and industry reports to develop a crime script, a template for committing a particular crime, for CNP fraud targeted at online retail businesses. Our crime script builds on the existing literature by outlining the practical steps bad actors take in committing the offence and using industry reports to identify the types of businesses and products most frequently victimised to understand the target selection process. We outline points of disruption at each stage of the script.

## Background

### Card-not-present fraud

As mentioned previously, CNP fraud involves the illegal acquisition and unauthorised use of another individual’s payment details for a CNP transaction. The payment details can be acquired through several methods, but these typically all begin with a data breach (Peretti 2009). Data breaches are, themselves, the result of unauthorised access (generally an attack of some form) or unintentional exposures (a ‘leak’) of personally identifiable information (PII), which can comprise information related to the cyber or physical world. The level of PII can include basic payment card payment details [including the card number, expiration date, cardholder’s name, and the card verification value (CVV)]. It may also involve what is known as “fullz”, which include additional verification details such as the cardholder’s date of birth, home address, and email and phone contact details (van Hardeveld et al. 2016). PII can also include login information for various accounts an individual might hold, including retail-specific accounts or alternative payment method accounts (e.g. PayPal). Large scale data breaches more commonly target financial information such as payment card details through sophisticated hacking approaches. Other common techniques are phishing or skimming (Peretti 2009). Bad actors can commit CNP fraud via an account takeover, using saved payment details or using payment card details to initiate a CNP transaction depending on the type of PII obtained. Bad actors also sell PII on dark web marketplaces (Wang et al. 2022).

Chargebacks are another critical issue that, while not directly involved in CNP fraud, can be a source of information for retailers about CNP fraud attacks, as well as an extra cost associated with these. Chargebacks are a consumer protection mechanism where a payment card company or bank can initiate the reversal of a merchant transaction on behalf of an account holder if they do not recognise, or dispute a transaction as being legitimate (Chargeback Gurus 2021; Big Commerce N.D.). This process is designed to protect cardholders from the cost of fraud, and banks can reverse a transaction without the merchant’s approval. In addition to the lost product and payment, merchants can also be charged a chargeback fee by banks, further adding to the cost associated with the fraud. While a chargeback can notify a merchant of a genuine fraud attack, the process can also be exploited by consumers, aka “friendly-fraud”, making chargebacks, at best, a proxy measure of CNP fraud (Chargeback Gurus 2021).

CNP fraud represents a significant challenge for businesses around the world. The impact of CNP fraud extends beyond the cost of loss of products for the business. Upon identifying unrecognised charges, the card issuer or original cardholder must initiate card cancellations and engage in efforts to recover the lost funds. Moreover, if a chargeback is issued, this generates additional costs to the targeted business or merchant. The company may also experience reputational damage as a result of targeted attacks. The associated costs of fraudulent transactions may also result in companies increasing prices to cover the increased cost of business or prevention measures, impacting legitimate customers.

### Crime scripts

Crime scripts provide a method of understanding a crime event in sufficient detail to identify key intervention points that, if targeted appropriately, can disrupt and prevent the crime from being committed (Leclerc 2017). Cornish (1994) applied the script process used in cognitive sciences to understand the component steps in actions, to understand the component steps in crime events. This approach fits within the broader framework of the rational choice perspective, which seeks to understand criminal decision-making throughout a crime event, arguing that the decision to commit a crime is based on a (bounded) rational weighing up of the perceived risks and perceived rewards (Cornish and Clarke 2017). Understanding the factors influencing individuals' decisions to commit a crime makes it possible to identify ways to increase the perceived risks or reduce the perceived rewards, making the crime event less likely.

While crimes are often thought of as a single event, from the perspective of a crime script, criminal acts can be seen as a sequence of events and associated decisions occurring in a particular order for an outcome. For most criminal acts, some actions and decisions need to be taken as preparatory steps before initiating the criminal act (e.g. gathering tools, equipment or recruiting associates). Equally, post-event actions are usually necessary for successful completion (e.g. disposal of goods, evading law enforcement). Crime scripts are typically broken down into acts or stages that are completed in order, the number of which can depend on the complexity of the crime. Each of these stages is then articulated with details of the knowledge and resources required, the specific actions offenders need to take, where they occur, who is involved, and any decisions made throughout (Chainey and Berbotto 2021; Cornish 1994; Dehghanniri and Borrion 2021; Van Nguyen 2021; Tompson and Chainey 2011; van Hardeveld et al. 2016).

The use of crime scripts by researchers has increased in the last decade. The type of crimes explored using the crime script approach has grown, including sexual offences (Leclerc et al. 2011), electronic waste (Tompson and Chainey 2011), drug manufacturing (Chiu et al. 2011), active shooter events (Osborne and Capellan 2017), fraud (van Hardeveld et al. 2016), and stolen data markets (Hutchings and Holt 2015). Moreover, this focus has expanded the utility of crime scripts beyond understanding and articulating the crime commission process and highlighting disruption points through crime prevention approaches (e.g. Chiu et al. 2011; Leclerc et al. 2011; Hao et al. 2015; van Hardeveld et al. 2016; Cook et al. 2019). Leclerc and Reynald (2017) even applied crime script analysis to guardian intervention of crimes in public locations and how prevention measures could facilitate intervention against the occurrence of crimes.

## Data

Due to the level of detail required, crime scripts will often draw on several sources of information, including interviews with offenders (Beauregard et al. 2007), survey data (Leclerc et al. 2011; Cook et al. 2019), court data (Peretti 2009; Chiu et al. 2011), incident reports (Beauregard et al. 2007; Hao et al. 2015; Van Nguyen 2021) and police statistics (Van Nguyen 2021). Interviews are considered a strong data source for crime scripts as they provide an opportunity for highly detailed information collection. However, they can be difficult for specific crime types with low incidence or low detection/solve rate.

CNP fraud represents a challenging crime type on which to collect comprehensive data. Bad actors often reside outside of the jurisdiction they offend in and take multiple steps to protect their anonymity, both of which provide barriers to detection and conviction. This presents a challenge to performing interviews to develop a crime script. Instead of this, several researchers have accessed dark web forums and marketplaces to view tutorials and information provided on a range of offences, as well as gain an understanding of how these marketplaces operate (Holt 2013; Hutchings and Holt 2015; Hao et al. 2015; van Hardeveld et al. 2016). The retail industry has several non-profit organisations heavily involved in providing risk management, fraud prevention, and loss management advice and conducting independent research [e.g. Loss Prevention Research Council (US), ECR Retail Loss (EU)]. These bodies, through working groups, collate information from retailers across sectors, establish best practices and coordinate solutions to problems facing their constituents.

In the current study, we draw on previous research and reports providing various crime scripts for payment card fraud, carding, computer fraud, and reshipping scams to populate the actions and steps of the crime script for CNP fraud. Our script is supplemented with information from retail and fraud prevention industry reports detailing the incidence rates of CNP fraud in different businesses, the types of products at greater risk of fraud, and some of the features of fraud detection systems to understand the decision-making processes involved.

## CNP fraud crime script

**Fig. 2:**
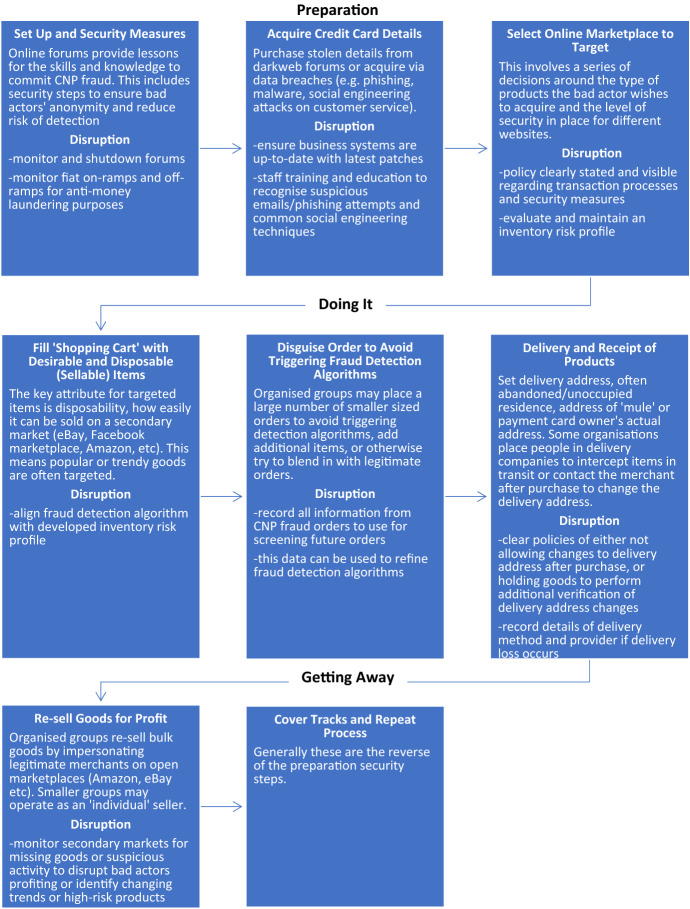
CNP fraud crime script with points of disruption

There are several stages involved in committing CNP fraud. Bad actors must prepare before the fraudulent transaction to increase the likelihood of success. Post-transaction, there are steps taken that realise the benefit without being identified or apprehended. As a result, the crime script that has been developed breaks CNP fraud into three distinct phases: preparation (prior actions), doing it (the transaction itself and receipt of goods), and getting away (after actions). The full script and points of disruption are demonstrated in Fig. 2.

### Preparation

The preparation stage of CNP fraud primarily focuses on two areas. The first is bad actors ensuring they have the required resources, particularly the necessary knowledge and equipment for the offence. The second is the offence-specific preparations. The basic equipment requirements of online fraud are low, needing only a computing device and internet access, providing few barriers to offending (Hutchings and Holt 2015; van Hardeveld et al. 2016). The knowledge and technical skills provide the main barrier to entry, with many bad actors overcoming this via tutorials available in dark web marketplaces and online forums. These marketplaces serve as important hubs, providing networking opportunities, tutorials and advice on methods, and access to vendors buying and selling stolen payment card details (Holm 2017; Hutchings and Holt 2015). The first time an individual accesses one of these marketplaces, they may need to create an account and undergo some security check or “vouch” process to prevent access by law enforcement agencies. The structure and security/reputation processes of these markets are discussed in detail by Holt and Lampke (2010) and Holt (2013). These marketplaces may provide basic tutorials for a range of online offences for free. Still, the more advanced tutorials and training require payment, with most marketplaces utilising forms of cryptocurrency as the preferred method.

After ensuring access to equipment and the requisite knowledge, bad actors begin the offence-

specific preparations. The most important preparation step in the CNP script is the security measures bad actors take to reduce the risk of detection by authorities (Hutchings and Holt 2015). There is a range of measures bad actors can take that provide varying levels of anonymity and security. At the basic level, steps such as clearing browser cookies before an offence, connecting to open/unsecured Wi-Fi networks, using The Onion Router (TOR) browsers, and using a virtual private network (VPN) will assist in increasing anonymity (Hutchings and Holt 2015; van Hardeveld et al. 2016). More comprehensive steps can involve using SOCKS5 proxies, using virtual encrypted disks, media access control (MAC) address spoofing, or the use of remote desktop computers and servers (Hutchings and Holt 2015; van Hardeveld et al. 2016). These security measures serve a range of functions, including masking (or faking) the physical location, anonymising the traffic, and protecting the physical computer itself through encryption if law enforcement does raid the location.

After ensuring their security, CNP bad actors need to acquire the payment details for the transaction. There is a range of ways this can be achieved depending on the resources and expertise of the individual. A detailed description of these methods is beyond this article's scope and would constitute a crime script itself, there is an extensive literature on identification theft in general (Copes and Vieraitis 2009a, b; Holm 2017; Vieraitis et al. 2015). Payment details are typically stolen via data breaches through hacking, phishing attacks, identification theft, social engineering techniques to trick customer service representatives into providing access, or use of staff inside a business (Peretti 2009; Hutchings and Holt 2015; van Hardeveld et al. 2016; Van Nguyen 2021). These stolen payment details can be used by the people that stole them, but often they are sold by vendors on dark web marketplaces (Hutchings and Holt 2015).

Once the bad actor has secured the payment details, they can begin the process of target selection: finding a suitable online store for the offence. Selecting an appropriate target involves a series of decisions weighing up the type of products sold, the security measures in place, whether an account is required, company policies, and the likelihood of merchants reporting to authorities if it is detected. Juniper Research (2020) reported that, with respect to retail, computer and electronics retailers accounted for 13% of detected fraudulent transactions, followed by general retail (9%) and clothing (5%). The Fraud Attack Index compiled by Forter and Merchant Risk Council (2016) ranked the apparel sector as experiencing the highest proportion of fraudulent attacks (both successful and unsuccessful), averaging $8.16 out of every $100 of US sales, and $14.45 in every $100 for international sales at risk of fraud. Considerations of the security measures in place can include broad heuristics such as “smaller [retailers] ones will have less security procedures in place” (van Hardeveld et al. 2016, p. 3) to specifically checking for things such as Verified by Visa, secure customer authentication (SCA; two-factor/multifactor authentication), or Payment Card Industry Data Security Standards (PCI-DSS). Company policies regarding deliveries may also factor into these considerations, such as parcel tracking, policies regarding the redirection of deliveries, etc. Once bad actors have selected the online business to target, they move into the second stage of the script, the transaction itself.

### Doing it

This stage involves selecting the desired product(s), minimising the risk of triggering fraud detection systems by disguising the order, the transaction itself, and the delivery and receipt of the product(s). An optional step that may be taken initially in this stage of the script can involve the creation of an account with the targeted website linked to an email address of the cardholder’s name (either compromised or created by the bad actor). This is known as new account fraud (see Malphrus 2009) and is, itself, a distinct crime commission process but one beyond the scope of this study.

The driving step in this stage of the script is the selection of the desired products, which are the means of how the bad actor benefits. There can be competing considerations for this decision. Of primary concern is how easily the bad actor can on-sell the product. If a product is in high demand, it can be sold quickly, for a greater profit, while reducing the time needed to store the product. As a result of this, popular brands or products experiencing a high demand (or low availability) are frequently targeted. Some recent examples of these high demand/low availability products include gym equipment during the early stages of COVID-19 lockdown (Lourenco 2020b) and new generation video game consoles (Clare et al. 2022). A report by Riskified (2016a) identified that within the apparel space, watches (10%), sneakers (6%), and jeans (3%), experienced higher fraud rates than most other items. Certain brands within a category also demonstrate different levels of desirability. Sneaker brands such as ‘Nike Lebron’ and ‘Timberland’ experienced the highest fraud rates at just over 40%, while brands such as ‘Converse’ and ‘Asics’ were around 10% (Riskified 2016a). Within electronics, high-risk items often include smaller, portable electronic devices such as smartphones, tablets, and action cameras (Ethoca N.D.).

The uneven distribution of CNP fraud across products is consistent with theft research and the concept of hot products and the CRAVED model (Clarke 1999). High-risk hot products tend to be Concealable, Removable, Available, Valuable, Enjoyable, and Disposable (Clarke 1999). Online markets such as eBay, Amazon, and Facebook Marketplace provide bad actors with opportunities to on-sell stolen products at scale (Newman and Clarke 2011). With this in mind, the disposability of a product is particularly salient in understanding the hot products of CNP fraud (Newman and Clarke 2011).

In conjunction with filling their ‘shopping cart’ with the desired products, bad actors will often take steps to disguise their order to appear legitimate to avoid triggering fraud detection systems. Broadly, the more items in an online order ‘cart’, the higher the risk of the order being fraudulent (Riskified 2016a, b, 2017). This is not a clear linear relationship. Riskified (2016a) found that luxury fashion orders of six or more items were ten times more likely to be fraudulent than single item orders, but orders including five items were less risky than those featuring four items.

Bad actors also try to blend into legitimate shopper traffic. Previous research identified recommendations in CNP fraud tutorials that individuals be aware of the local time for the businesses being targeted or the physical location they have set their computer to appear to be in (van Hardeveld et al. 2016; Hutchings et al. 2019). Matching the standard shopping hours for the location is a low-effort way to reduce the transaction appearing unusual and being flagged for review.

In addition to blending in with shopping time patterns, offenders tend to adapt with shopping trends. With the impact of COVID-19 on travel and working from home, there was some evidence that CNP bad actors rapidly adapted their decision-making, shifting from a small number of large ‘shopping carts’ with high-value electronics such as cameras and other devices, to targeting office supplies with lots of orders but smaller ‘shopping carts’ (Townsley and Hutchins 2021). Bad actors may also spread fraudulent transactions across several businesses, or put through several smaller transactions instead of one large transaction (Lourenco 2020a; van Hardeveld et al. 2016). There have been suggestions that fraud activity spikes during holiday periods and major sales events (such as Black Friday) however this is disputed (Hutchings and Holt 2015). Riskified (2016a) suggest that while data may suggest an increase in fraudulent activity in these times, this is due to an increase in false positives, rather than true fraud attempts. Extreme transaction volumes can overwhelm fraud prevention measures which can undermine the ability to review flagged orders, which, in turn, increases the chance that legitimate orders get rejected. As an example, across Black Friday and Cyber Monday, 75% of transactions a sample of luxury fashion merchants flagged as fraudulent were found to be false positives, three times the average false positive rate across the rest of the year (Riskified 2016a).

Once bad actors have selected the items and taken steps to disguise their order, they need to complete the transaction with the stolen payment details and set up delivery. There are several delivery methods that can be used to minimise risk to the bad actor. At the most basic, this may be a drop location unrelated to the bad actor’s personal life that they, or a money mule, can collect the package from, such as an abandoned property or post office collection (Peretti 2009; van Hardeveld et al. 2016; Van Nguyen 2021). In some cases, the cardholder’s actual address may be provided as the delivery location to lower the fraud risk score. Once the transaction is approved, the bad actor will call the merchant or customer service team and have the delivery address changed (Lourenco 2020a). This can often involve a socially engineered script based on the business’ own policies to manipulate the customer service employees (Lord 2020). Organised crime groups may place people working for the shipping companies to intercept and redirect the fraudulent orders (Lourenco 2020a).

One of the more common methods involves the use of reshipping mules (Hao et al. 2015; Van Nguyen 2021). In this method, the bad actor may use a reshipping scam website to hire a (sometimes unsuspecting) mule to receive the package and reship it to the bad actor (Hao et al. 2015; Van Nguyen 2021). Using this method, a bad actor located internationally can have the fraudulently purchased products delivered to an address close to the original cardholder’s location and then reshipped outside the jurisdiction to decrease the risk of being caught (Hao et al. 2015; Van Nguyen 2021). The mule is informed of when to expect the package, often addressed to the original cardholder, and often sent a prepaid shipping label to reship the package. Once it arrives, the mule repackages the products, attaches the shipping label and ships the products to a drop site located in the same city as the original CNP bad actor (Hao et al. 2015; Van Nguyen 2021). At this point either the bad actor may collect the package, or it may be retrieved by a local mule and then delivered to the bad actor (Hao et al. 2015; Van Nguyen 2021).

Using industry information, Riskified (2017) found that different shipping methods are associated with different levels of fraud risk, with ground-based shipping (which is typically cheaper, but slower) approved 98% of the time, while express shipping methods (more expensive, but faster) were approved 89% of the time. This is likely due to the fact the higher shipping cost is not actually being paid by the bad actor, so the convenience of the package arriving sooner with express delivery does not have a penalty (Riskified 2017).

### Getting away

The final stage of the CNP fraud crime script involves two main components: post-offence security steps, and steps to profit from the fraud. The post-offence security steps are typically the reverse of the preparation security steps, such as clearing cookies from web browsers, disconnecting VPNs, proxies, erasing virtual encrypted disks and so on. The bad actor may also leave a review on the dark web forum for the individual the card details were purchased from, or other general reputation-management steps (Holt and Lampke 2010; Hutchings and Holt 2015; van Hardeveld et al. 2016). Finally, the bad actor must take steps to profit from the offence by selling the purchased goods. Second-hand retail marketplaces such as eBay, Facebook Marketplace, or Amazon provide ample opportunity for this (Aniello and Caneppele. 2018).

## Points of disruption/prevention

Situational crime prevention (SCP) uses a detailed understanding of a particular crime problem to find ways to alter the situation or circumstances in which the crime occurs (Clarke 1995, 2017). By analysing the CNP fraud crime script, key steps can be identified that, if targeted, have the potential to disrupt the crime from being successfully committed. The recommended measures can be implemented by a range of stakeholders, including law enforcement agencies, retail businesses, financial institutions, and industry regulators.

Arguably the most significant disruption would be achieved by preventing bad actors from obtaining the payment details or account details for takeover. While there are several ways that bad actors obtain this information, prevention strategies all revolve around target hardening. This involves technology measures such as ensuring security systems have the latest patches for vulnerabilities, and staff and customer accounts use adequate security such as multifactor authentication (MFA) and strong password guidelines (Akram and Ping 2019; Lord 2020). It is also important to incorporate staff training in recognising phishing attempts and suspicious emails, awareness of social engineering techniques, and what processes should be followed in these situations (Jampen et al. 2020; Lord 2020). As the technological security measures improve, the human component can become the weakness that bad actors will exploit. If target hardening measures do fail and businesses identify a data breach, steps should be taken to deny the benefits. This should involve a rapid response to notify affected customers and relevant financial/regulatory bodies in addition to any mandatory reporting requirements, such as the intent of legislation in many countries [e.g. the Privacy Amendment (Notifiable Data Breach) Act 2017 (Australia); General Data Protection Regulation (European Union); Privacy Act 2020 (New Zealand); Data Breach Notification Law (US)].

Law enforcement agencies are continually engaged in efforts to identify and shut down dark web forums and marketplaces. While these efforts have had successes, such as the shutdown of Silk Road in 2013 or DarkMarket in early 2021, often this results in users migrating to another marketplace (Ladegaard 2019; Wang et al. 2022), making long-term impacts difficult with this strategy. A similar challenge faces efforts to trace and analyse the blockchain of cryptocurrencies. As the tools for blockchain analysis improve, there can be a shift to new cryptocurrencies which circumvent these, such as Monero which takes measures to prevent the tracing of transactions.

Businesses can implement strategies to reduce the likelihood of being targeted by CNP bad actors as well. Clearly displaying the transaction, shipping, and return policies up-front (or included in any receipt provided) and security measures in place can be a selling point to customers as well as a deterrent to potential bad actors that the website is not a suitable target (Revel Systems 2021; Mitchell 2022). These measures can also provide evidence to dispute chargebacks that may arise (Worldpay Editorial Team 2019).

In addition to policy measures, there are some fraud detection methods that can be implemented prior to the transaction stage to detect activity that can be indicative of a higher risk of fraud. This can include customer behaviour monitoring, device fingerprinting, and onboarding processes during account creation, such as address verification, multifactor authentication, and data enrichment (U.S. Payments Forum 2020; Kadar N.D.). Customer behaviour monitoring evaluates patterns of behaviour in how a customer interacts with the retail platform and can be used for early identification of bots, bad actors performing card testing (checking if the card is active before making larger purchases), and patterns of page navigation indicative of higher fraud risk (U.S. Payments Forum 2020). Device fingerprinting collects information related to the device being used to interact with the retail platform and can be used to identify inconsistencies between account records, entered information, and details of the device including (but not limited to) the IP address, operating system, time zone, and language (Kadar N.D.). The onboarding process can provide customer information that enables further checks and authorisations in future if suspicious activity is identified. Collecting a billing address can enable address verification (AVS) with the card issuer to see if they are a complete match, partial match, or no match (Kadar N.D.). If an email address and phone number is collected, multifactor authentication steps can be added for verification and authorisation, while also providing avenues for data enrichment (U.S. Payments Forum 2020; Kadar N.D.). Data enrichment checks collected information against external sources such as social media accounts registered to the email address, country in which the phone number is registered, all of which can allow the detection of discrepancies which could indicate an illegitimate user (Kadar N.D.). Measures such as these all provide information that when used in concert with other layers of security can increase the likelihood of identifying bad actors early and preventing a fraudulent transaction.

Another key step in the CNP fraud crime script is the transaction itself. While there is little external agencies can do at this point in the process, there are a number of strategies retailers can enact for the purpose of target hardening or denying benefits. The first side of this, target hardening, focuses on the use of strong, well-designed fraud detection algorithms. These would usually be provided by a commercial, third-party, who incorporate a myriad of data sources, some external to an individual retailer, to provide signals at a transaction level. These signals can be used to create rules that permit acceptance or manual review (Knuth and Ahrholdt 2022).

Visa (2013) identified features that can indicate a higher risk of fraud which include:

- Larger than normal orders
- Orders with multiples of the same product
- Orders originating overseas
- Inconsistencies between order details, such as different shipping and billing addresses
- Multiple payment cards originating from the same IP address
- Multiple orders with the same shipping address but different payment details
- Multiple orders with the same payment details but several different shipping addresses

In addition to these general features of risk, businesses should enhance fraud detection algorithms with their own transaction data and inventory risk profiles. By augmenting the generalised fraud detection algorithms with in-house developed risk profiles businesses increase their ability to detect fraud patterns that may be specific to them. As a component of this, online retailers should record information associated with both detected and prevented CNP fraud orders, as well as those that are approved and detected after the fact. Details regarding contact information, delivery address and method, payment details, and the items ordered are all valuable pieces of information that can be used to screen future orders for review. These measures would counteract instances of repeat offending where bad actors return to websites they have previously had success targeting, a trend which demonstrated increases of 66% across 2019–2020 (Forter 2020).

Strategies for monitoring delivery methods can be difficult to implement due to the range of methods that might be employed and the ability of bad actors to circumvent prevention efforts. For example, businesses can implement policies limiting the ability of bad actors to contact customer service to change a delivery address after the transaction is approved, but third-party delivery companies sometimes allow customers to reroute, or change the delivery address without contacting the seller. Similarly, parcel tracking numbers can prevent bad actors from intercepting a package before it arrives at the listed delivery address, but with the adoption of no-contact delivery during COVID-19 restrictions and the option for packages to be left in a safe place, bad actors can still intercept these from the address (Lourenco 2020a). The main way organisations can combat this stage is by benchmarking any losses relative to both the delivery method, the delivery provider, and industry peers. This process can identify flaws in delivery processes, riskier delivery methods, or providers associated with higher-than-average losses.

The final intervention point in the CNP fraud script centres around denying benefits to the bad actors. In order to profit from the fraud, the bad actor needs to be able to sell the fraudulently purchased products for more than the initial cost of the payment details. A detailed understanding of the stolen goods market (e.g. Sutton 2010) can provide insight that would help retailers protect their inventory and make CNP fraud more difficult and less desirable to commit. In addition to identifying patterns that can be incorporated into fraud detection algorithms, this information may provide businesses and law enforcement agencies opportunities for product recovery and arrest of bad actors. This, however, may only be possible with strong, constructive relationships with the major third-party selling platforms. Increased collaboration between the various stakeholders (retailers, law enforcement, secondary marketplaces, and financial institutions) would help facilitate the sharing of intelligence on the full CNP script, rather than siloing information on each stage to only a few stakeholders directly involved (Levi 2003).

## Discussion and conclusions

The current paper focused on CNP fraud that involves the illegal acquisition and unauthorised use of another individual’s payment details to engage in online transactions. The volume and prevalence of this crime type in recent years have exploded with CNP fraud now one of the most common types of fraud perpetrated in the cyber environment. Given the seemingly unabated rise in CNP fraud, more needs to be done to prevent the crime from occurring and to reduce victimisation rates. To contribute to the discussion around what more can be done to reduce and prevent CNP fraud, the current paper used a crime script approach. First, we sought to analyse the steps involved in the commission of this crime and second, identify potential areas of disruption through specific crime prevention strategies.

Providing a detailed description of the actions taken within each of the steps of preparation (prior actions), doing it (the transaction itself and receipt of goods), and getting away (after actions) is critical to determining specific and potentially more effective and impactful strategies for crime prevention. The micro-actions undertaken by bad actors might be easily overlooked given that a cursory examination of this crime type could lead to the conclusion that the fraud appears to take place almost instantaneously through online transactions. By applying crime script analysis, our paper was able to identify the micro-actions within each phase of the crime script that led to the generation of crime prevention strategies. The benefit of crime script analysis is that crime prevention strategies are able to be generated for each phase of the crime commission process, so multiple intervention points are identified and a wide scope of stakeholders who may be able to action prevention activities are able to be identified. We were able to determine that several stakeholders including law enforcement agencies, retail businesses, financial institutions, cyber security firms and industry regulators could all impact CNP fraud prevalence, detection and prevention.

Moving from the application of crime script analysis, crime prevention strategies generated using a SCP approach were discussed. The most significant crime prevention point, in that it would stop the crime at the very initial stages of the commission process, was preventing bad actors from obtaining the payment details or account details for takeover. Target hardening, involving preventing bad actors from obtaining PII in the first place, through strategies such as ensuring security systems have the latest patches for vulnerabilities, using security such as multifactor authentication (MFA) and strong passwords. This involves a shared responsibility by potential victims and organisations to ensure information is secure. Law enforcement has a role to play, specifically, it was identified their efforts in shutting down dark web forums and marketplaces.

Most of the crime prevention strategies that were identified were within the scope of control of businesses and particularly, retailers. This might involve deterring bad actors through the promotion of clear transaction policies and security measures that may deter bad actors from attempting to commit CNP fraud. Businesses can also take proactive steps by developing fraud detection algorithms to identify potentially fraudulent transactions as early as possible in the crime commission process. Further along the crime commission process, businesses may be able to identify possible frauds and in turn, develop more proactive identification measures by examining delivery methods used by bad actors. Retailers should seek to monitor themselves and develop collaborations with platforms that host secondary markets. This strategy will curb the ability of bad actors to offload the goods that are obtained through CNP and reduce the rewards that are able to be gained from engaging in this type of crime.

Like any study focusing on human behaviour involving deception, the description contained in this article will be incomplete. The mix of academic and grey literature suggests this problem is a fast-evolving phenomenon that may have an entirely different composition in a short time in the future. Additionally, we have not used accounts of bad actors themselves to contextualise our findings. The scope of this paper has also limited the applicability of the findings of this article. Informed by the crime script approach, we have focused on the instrumental steps required for the successful commission of CNP fraud in our treatment, which may have limited the types of prevention measures outlined here. Nevertheless, we hope the application of the crime script, breaking down a crime event into stages and considering points of disruption at each step, provides an illustration for professionals, policymakers and law enforcement for ways of thinking about defending their businesses or disrupting bad actors.

As CNP fraud maintains its upward trajectory, we must continue to work towards better understanding how this crime is successfully enacted, what our motivations for bad actors that can be curbed to make it a less attractive and easy crime to commit and how to work across multiple stakeholders who all have a role to play in crime reaction and crime prevention. This paper, using a crime script analysis methodology clearly articulated the micro-actions or activities involved in CNP fraud. In turn, we were able to identify intervention points that may be leveraged to reduce CNP fraud. This paper provides an important contribution to clearly articulating the processes involved in CNP fraud and points to some recommendations that can be used for better crime prevention outcomes.
